# Indicators of maternal anxiety, stress, and depression and developmental outcomes in preterm children: A systematic review

**DOI:** 10.1002/imhj.70099

**Published:** 2026-05-14

**Authors:** Bianca Basso Rustiguelli da Silva, Cláudia Maria Gaspardo

**Affiliations:** ^1^ Department of Neurosciences and Behavior Ribeirao Preto Medical School, University of São Paulo São Paulo Brazil

**Keywords:** maternal anxiety, maternal depression, maternal stress, prematurity, trajectories of development

## Abstract

The study was conducted in Brazil and aimed to systematically review empirical research examining the association between maternal indicators of anxiety, stress, and depression and the developmental outcomes of preterm children. A literature search was conducted across PubMed, Web of Science, PsycINFO, EMBASE, Scopus, LILACS, and SciELO. Sixteen studies were analyzed. The systematic review revealed a significant association between maternal anxiety, depression, and stress indicators and poorer developmental outcomes for children born prematurely. This association was observed whether it was considered a single indicator of maternal mental health or a combination of two or more indicators. The systematic review affirms the impact of maternal mental health on the development of preterm children in early childhood.

## 1 INTRODUCTION

The global average for preterm births, which refers to those born at less than 37 weeks of gestation, is 11%. This percentage corresponds to an estimated 15 million neonates worldwide (Walani, 2020). Preterm birth, along with child morbidity and mortality, presents significant challenges in the health and child development fields. As infants navigate the initial survival challenges, it becomes crucial to focus on mitigating damage and long‐term effects on their developmental trajectory, given their vulnerable state (World Health Organization, 2023).

Preterm birth can lead to several adverse effects on a child's health due to their neonatal condition. These effects include brain damage, particularly peri‐intraventricular hemorrhages, which can severely impact the central nervous system (Wu et al., 2020). Additionally, preterm infants are at an increased risk of developing cerebral palsy (Soleimani et al., 2020), respiratory complications like pulmonary bronchodysplasia (Kline et al., 2019; McGowan et al., 2022), intestinal issues such as necrotizing enterocolitis (Gregory et al., 2011), and sensorineural impairment (Hee Chung et al., 2020).

The developmental trajectories of preterm children have been thoroughly studied. The research indicates a higher likelihood of experiencing challenges in various neurodevelopmental areas (Rodrigues et al., 2021), particularly delayed motor development (Filippa et al., 2019; Lee et al., 2016; Miyagishima et al., 2016), cognitive deficits (Capobianco & Cerniglia, 2017; McGowan et al., 2022; Nobre et al., 2019), and behavioral and emotional difficulties (Cassiano et al., 2016; Cassiano et al., 2019).

Due to the circumstances surrounding preterm birth and the necessary intensive care, early separation between the mother and preterm neonate is often difficult to avoid. As a result, preterm neonates are typically deprived of physical contact with their mothers during the early stages of development, with opportunities for contact occurring only during visits to the Neonatal Intensive Care Unit (NICU). In this context, certain specificities pertain to the initial interaction between a mother and her child. As the neonate needs to remain in the incubator most of the time or can present clinical instability, skin‐to‐skin contact, cuddling in the mother's lap, and even breastfeeding are less common, despite being part of the developmental care for preterm infants (Baylis et al., 2014). To address these challenges at the beginning of life, family‐centered care interventions have been increasingly adopted in NICUs worldwide. These developmental care approaches encompass practices such as skin‐to‐skin care, the implementation of single‐family rooms or single‐bay ward models, information‐sharing interventions, and the promotion of parent participation, which have been associated with improvements in parent‐infant bonding, parental mental health, and better neonatal outcomes. Evidence indicates that family‐centered care interventions increase parental engagement, enhance satisfaction, and reduce stress, anxiety, and depressive symptoms among caregivers, while also promoting infant weight gain, adherence to breastfeeding, and a shorter hospital stay (Hodgson et al., 2025; Lean et al., 2018).

Additionally, preterm neonates may be less responsive to environmental stimuli (Schiavo & Perosa, 2020) or hyperresponsive to external stimuli (McGowan et al., 2022), which can challenge their initial interactions with their mothers. It is essential to achieve adequate sensorineural and emotional development in the neonate by optimizing both the macroenvironment (e.g., lighting and sound) and the microenvironment, including proper postural control, effective pain management, and minimal manipulation (Gómez‐Cantarino et al., 2020). Therefore, mothers need to learn to regulate the stimuli they provide to their children to avoid disrupting their clinical stability and thereby affecting their development. This, in turn, can make the initial experiences of mother‐infant bonding less spontaneous and more insecure. Mothers also have to contend with their dependence on medical and nursing care, temporarily adopting a passive role in caring for their children (Palmquist et al., 2020), which can further exacerbate negative feelings associated with motherhood (Fowler et al., 2019).

**KEY FINDINGS**

- There is a relationship between maternal anxiety, depression, and stress and the poorer developmental outcomes of preterm children during early childhood.
- Indicators of anxiety, depression, and maternal stress collectively heightened the risk of negative outcomes, primarily in the behavioral domains of preterm children.
- Indicators of anxiety or depression significantly impacted neurodevelopmental and behavioral/emotional indicators in preterm children.

**STATEMENT OF RELEVANCE TO INFANT AND EARLY CHILDHOOD MENTAL HEALTH**

Child mental health is closely linked to maternal mental health. Therefore, studying maternal mental health indicators among mothers of preterm children is a critical area of research for child development and mental health. This allows for the identification of risk factors affecting developmental trajectories and the promotion of maternal and child mental health.

The experience of having a preterm birth followed by hospitalization in a NICU can be very challenging for mothers, even when they are healthy (Field, 2017; Fowler et al., 2019). The stress and anxiety that mothers of preterm neonates face can make it challenging to care for their children (Anderson & Cacola, 2017; Field, 2017; Yates et al., 2022). It can also affect the establishment and strengthening of the parent‐child bond (Anderson & Cacola, 2017; Field, 2017). The emotional implications of preterm birth can be pretty significant, which can then increase risk factors and negatively impact the child's developmental outcome (Ionio et al., 2019).

During the first year after birth, a mother's mental health can significantly affect her child's development; the foundations of integral growth are closely tied to the relationship established by the mother‐infant dyad. Maternal behaviors such as intrusiveness and apathy, often seen in cases of depression, anxiety, and stress, can disrupt the mother's interaction with her child and negatively impact their development (Schiavo & Perosa, 2020). Preterm birth is a risk factor for maternal anxiety, which in turn can negatively affect the infant's breastfeeding and sleep patterns, the dyad's bonding and interaction, and indicators of the child's development and behavior through adolescence (Field, 2017).

It is worth noting that depressive symptoms can persist after preterm birth (Gerstein et al., 2019; Liu et al., 2017). These symptoms may even persist during the neonate's stay in the NICU (Gerstein et al., 2019; Trumello et al., 2018) and can linger in the short‐ and medium‐term following discharge (Kantrowitz‐Gordon et al., 2016; Liu et al., 2017; Pace et al., 2016). Importantly, the early signs of depression can have a significant and lasting impact on both mothers and their children. According to research, 20% of mothers who show signs of depression when their infant is discharged from the NICU continue to exhibit clinical indicators of depression for up to 5 years after giving birth prematurely; this can lead to a lack of maternal responsiveness and affect the quality of the mother‐child relationship (Gerstein et al., 2019).

Furthermore, Gray et al. (2018) found that mothers of preterm infants experienced higher levels of stress compared to mothers of full‐term infants. Additionally, preterm children displayed more internalizing and externalizing behavior problems at 4, 12, and 24 months of age than their term counterparts. The study also indicated that the behavior problems identified in preterm children were associated with elevated levels of maternal stress.

Given the points mentioned above, the care of premature infants admitted to the NICU must also include assessing the mental health of their mothers, along with implementing psychological intervention strategies. This will help activate protective mechanisms against negative maternal emotions and promote healthy bonding between mother and child. There remains a gap in the literature regarding the impact of the three indicators of maternal mental health (stress, depression, and anxiety) on the developmental outcomes of preterm children. Although studies exist in the current literature that examine isolated indicators, such as depression (Aoyagi & Tsuchiya, 2019), they do not exclusively consider the group of preterm infants. The present study aimed to systematically review empirical studies that assessed the association between maternal indicators of anxiety, stress, and depression and the developmental outcomes of preterm children.

## 2 METHOD

The current review has been registered in the PROSPERO database (blinded for review).

### 2.1 Search strategy

This systematic review was conducted according to the Preferred Reporting Items for Systematic Reviews and Meta‐Analyses (PRISMA; Page et al., 2021). The review involved searching the PubMed, Web of Science, PsycINFO, EMBASE, Scopus, LILACS, and SciELO databases using the following search strategy: (maternal anxiety) OR (maternal depression) OR (maternal stress) OR (parental stress) AND (prematur*) OR (trajectories of develop*). The search took place in April 2024.

### 2.2 Selection criteria

The inclusion criteria were: (i) studies examining the associations between maternal indicators of anxiety, depression, and stress and the developmental outcomes of preterm children; (ii) studies published from 2013 to 2024; (iii) empirical observational studies employing quantitative methodology; (iv) studies published in English, Portuguese, or Spanish. The exclusion criteria were: (i) review studies, meta‐analyses, editorials, protocols, case studies, abstracts published in the proceedings of scientific events, book chapters, and theoretical‐conceptual studies; (ii) studies conducted during pregnancy; (iii) studies that did not assess maternal mental health indicators; (iv) studies that did not evaluate developmental indicators for preterm children; (v) studies that failed to associate maternal mental health indicators with the developmental outcomes of preterm children; (vi) studies that included samples of both fathers and mothers; (vii) studies with samples of adolescent mothers; (viii) studies with samples of non‐biological mothers; (ix) studies that did not specify maternal age; (x) studies with samples that did not include preterm toddlers.

### 2.3 Selection of studies

**FIGURE 1:**
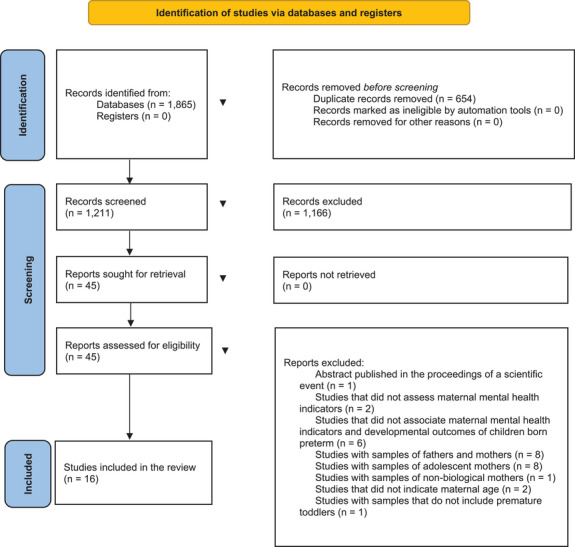
Flowchart of the search for the studies.

Figure 1 illustrates that 1865 articles were identified in the referenced databases. First, 654 duplicate articles were excluded because they were indexed in more than one database. Second, 1211 records were screened, and their titles and abstracts were analyzed, resulting in the exclusion of 1166 articles through the systematic application of inclusion and exclusion criteria. Then, 45 remaining articles were fully read, resulting in the exclusion of 29 articles: (i) abstract published in scientific events (*n* = 1); (ii) studies that did not assess maternal mental health indicators (*n* = 2); (iii) studies that did not associate maternal mental health indicators with the developmental outcomes of preterm children (*n* = 6); (iv) studies with samples of fathers and mothers (*n* = 8); (v) studies with samples of adolescent mothers (*n* = 8); (vi) studies with samples of non‐biological mothers (*n* = 1); (vii) studies that did not indicate maternal age (*n* = 2); (viii) studies with samples that did not include preterm toddlers (*n* = 1). Finally, 16 articles were reviewed.

### 2.4 Data extraction

All studies were reviewed in accordance with the PRISMA checklist (Page et al., 2021), and key study characteristics were identified. Information extracted from the studies included the following topics: authors and year of publication, country, objectives, design, sample, instruments and measures, maternal mental health variable, preterm children's developmental outcome variable, data analysis, and the main findings of the studies. The first author read all articles and performed data extraction coding based on these items, which the second author later reviewed to ensure the accuracy of the analysis.

### 2.5 Methodological quality assessment

The methodological quality of the 16 studies was assessed according to the Strengthening the Reporting of Observational Studies in Epidemiology (STROBE) Statement (von Elm et al., 2008), with consensus reached by two independent researchers (the first and second authors). The STROBE statement comprises 34 items that cover the following topics: title and abstract; introduction (background, objectives); method (study design, setting, participants, variables, measurement, bias, study size, quantitative variables, data analysis); results (participants, descriptive data, outcome data, primary results, other analyses); discussion (key results, limitations, interpretation, generalizability); and other information (funding). The final score was the total of the 34 items, with a maximum score of 34 points; the higher the total score, the greater the methodological quality.

## 3 RESULTS

### 3.1 Overview of studies

The 16 studies reviewed included 2724 mother‐child dyads, with sample sizes ranging from 29 to 318 participants (averaging 144). The studies were conducted in nine different countries: the United States of America, England, Spain, Italy, Norway, Turkey, China, Hungary, and Israel, across three continents: Europe (62%), Asia (31%), and North America (7%). Regarding the study design, 12 studies (75%) employed a prospective longitudinal design, while 4 (25%) used a cross‐sectional design.

The sample of the studies included preterm children and their biological mothers over 18 years of age, with or without a control group of full‐term children. Most studies examined clinical samples of preterm children (69%), while a smaller proportion (31%) compared preterm and full‐term groups. The studies defined preterm children as those born before 37 weeks of gestational age (GA). They identified full‐term children as those born after 37 weeks of GA, which aligns with the World Health Organization (WHO) guidelines (World Health Organization, 2023). The preterm children had an average GA of 30 weeks, classified as very preterm according to the WHO classification (World Health Organization, 2023). Conversely, the full‐term children had an average GA of 40 weeks.

It is notable that out of the studies conducted, only two utilized the comprehensive WHO (World Health Organization, 2023) classification to identify the preterm sample, dividing it into four categories: late preterm (GA of 36 weeks), moderate preterm (GA of 32 to 36 weeks), very preterm (GA of 28 to 31 weeks), and extremely preterm (GA of less than 28 weeks). However, 16% of the studies categorized the samples as low birth weight (<2500 grams), very low birth weight (<1500 grams), and extremely low birth weight (<1000 grams) based on birth weight (WHO, 2023).

Considering the sociodemographic characteristics of the samples, it is essential to note that all included studies included at least one parental variable, such as age, educational background, household income, or marital status. However, there was considerable disparity in how these parameters were measured and analyzed. In the majority of the included studies, sociodemographic indicators were presented only as baseline descriptive characteristics and were not systematically reported. Mothers were most frequently in early or middle adulthood, with maternal age typically varying from the mid‐20s to early 30s (Montirosso et al., 2018; Santos et al., 2016). Educational status was commonly reported, with most mothers having completed at least high school and many having higher education in European and North American samples (Pisoni et al., 2020; Neri et al., 2020). In contrast, studies conducted in Asian and Latin American contexts more often included families with lower levels of maternal education and more diverse educational backgrounds (Santos et al., 2016; Wang et al., 2018). In studies reporting family income (Gao et al., 2023; Ren et al., 2024; Santos et al., 2016), most samples reported low‐ to middle‐income households. Other sociodemographic indicators, such as marital status, showed that most mothers were married or cohabiting, with single parenthood being less common but observed in some samples (Assal‐Zrike et al., 2021, 2022). It is essential to note that none of the studies listed reported data on the race or ethnicity of the samples. Taken together, these findings suggest that, although there is variability across cultural and geographic contexts, the predominant profile of participating was young to middle‐aged mothers with at least a high school education, often from low‐ to middle‐income backgrounds.

### 3.2 Maternal anxiety, stress, and depression

The 16 studies evaluated indicators of maternal anxiety, stress, or depression. The average age of the mothers at the time of assessment was 31 years. Eight studies examined multiple indicators of maternal mental health (Assal‐Zrike et al., 2021, 2022; Bozkurt et al., 2017; Kenyhercz & Nagy, 2020; Pérez‐Pereira & Baños, 2019; Pisoni et al., 2020; Santos et al., 2016; Zengin Akkus & Bahtiyar‐Sayganet, 2022), while eight studies focused on only one (Coletti et al., 2015; Gao et al., 2023; Kleine et al., 2020; Moea et al., 2016; Montirosso et al., 2018; Neri et al., 2020; Ren et al., 2024; Wang et al., 2018). Among the analyzed articles, the assessment of all three indicators of maternal mental health (Assal‐Zrike et al., 2021, 2022; Kenyhercz & Nagy, 2020) or maternal depressive symptoms alone (Gao et al., 2023; Moea et al., 2016; Montirosso et al., 2018; Ren et al., 2024; Wang et al., 2018) was more prevalent.

Regarding the instruments used for assessing maternal depression, the Edinburgh Postnatal Depression Scale (EPDS) was predominantly employed (Gao et al., 2023; Moea et al., 2016; Montirosso et al., 2018; Ren et al., 2024; Wang et al., 2018; Zengin Akkus & Bahtiyar‐Sayganet, 2022), followed by the Center for Epidemiological Studies Depression Inventory (CESD) (Assal‐Zrike et al., 2021, 2022; Pisoni et al., 2020; Santos et al., 2016). The most commonly used instruments for assessing maternal anxiety were the State‐Trait Anxiety Inventory (STAI) (Assal‐Zrike et al., 2021, 2022; Kleine et al., 2020; Pisoni et al., 2020; Santos et al., 2016) and the Beck Anxiety Inventory (BAI) (Bozkurt et al., 2017; Kenyhercz & Nagy, 2020). Lastly, the Parenting Stress Index—Short Form (PSI‐SF) was the most frequently used instrument for evaluating maternal stress (Coletti et al., 2015; Pérez‐Pereira & Baños, 2019; Zengin Akkus & Bahtiyar‐Sayganet, 2022), followed by The Parental Stress Scale: NICU (PSS: NICU) (Pisoni et al., 2020; Santos et al., 2016).

### 3.3 Developmental outcomes of preterm children

The studies assessed the development of preterm children at various stages. The most common age for evaluation was 12 months, adjusted for prematurity (Coletti et al., 2015; Neri et al., 2020; Pisoni et al., 2020; Ren et al., 2024; Santos et al., 2016). Notably, in three of the studies, participants were included at a range of ages (Bozkurt et al., 2017; Gao et al., 2023; Kenyhercz & Nagy, 2020; Kleine et al., 2020; Ren et al., 2024), while five articles focused on specific periods (Coletti et al., 2015; Montirosso et al., 2018; Pérez‐Pereira & Baños, 2019; Santos et al., 2016; Wang et al., 2018). Additionally, four studies examined children over a single period (Assal‐Zrike et al., 2022; Moea et al., 2016; Neri et al., 2020; Pisoni et al., 2020).

Regarding the age of assessment of preterm children development, the most studies (75%) corrected the age for prematurity (Bozkurt et al., 2017; Coletti et al., 2015; Gao et al., 2023; Kenyhercz & Nagy, 2020; Kleine et al., 2020; Montirosso et al., 2018; Neri et al., 2020; Pisoni et al., 2020; Ren et al., 2024; Santos e al. et al., 2016; Wang et al., 2018; Zengin Akkus & Bahtiyar‐Saygan, 2022) while 25% of the studies conducted their longitudinal assessments without correcting for the age of preterm children (Assal‐Zrike et al., 2021, 2022; Moea et al., 2016; Pérez‐Pereira & Baños, 2019).

Regarding the developmental outcomes assessed, there was a predominance of neurodevelopment indicators (Bozkurt et al., 2017; Coletti et al., 2015; Pisoni et al., 2020; Santos et al., 2016; Wang et al., 2018), behavioral and emotional indicators (Kenyhercz & Nagy, 2020; Kleine et al., 2020; Montirosso et al., 2018; Pérez‐Pereira & Baños, 2019), and social behavior (Assal‐Zrike et al., 2021, 2022; Moea et al., 2016). Five studies used multiple assessment instruments (Assal‐Zrike et al., 2021; Kleine et al., 2020; Montirosso et al., 2018; Moea et al., 2016).

In considering the instruments used to assess developmental outcomes, four studies utilized the Bayley Scales of Infant Development (Bozkurt et al., 2017; Coletti et al., 2015; Santos et al., 2016; Wang et al., 2018), while two articles employed the Griffiths Mental Development Scales (Neri et al., 2020; Pisoni et al., 2020), another two used the Child Behavior Checklist for Ages 1.5–5 (Montirosso et al., 2018; Pérez‐Pereira & Baños, 2019), and two utilized the Alarm Distress Baby (Assal‐Zrike et al., 2022; Moea et al., 2016), respectively.

### 3.4 Main findings of studies

Table 1 summarizes key findings from studies that evaluated one indicator of maternal mental health and developmental outcomes in preterm children.

**TABLE 1:** Main findings of the significant results of studies that assessed one indicator of maternal mental health and developmental outcomes in preterm children (*n* = 8).

Author (year) country	Study design/data analysis	Sample		Main findings
		Mothers	Children	
Neri et al. (2020) Italy	Longitudinal Study/Repeated Measures MANOVA	*n* = 147 Mean age 33 y Indicator of mental health: anxiety At 3 m (corrected age for preterm infant)	*n* = 81 full‐term healthy infants (>36 w) *n* = 41 infants VLBW (<32 w) *n* = 25 infants ELBW (<28 w) Developmental outcome: psychomotor At 3 m (T1), at 9 m (T2), and at 12 m (T3) (corrected age for preterm infants)	Anxious mothers: Infant locomotor development—VLBW at T1 ↑ than at T2 and at T3 Non‐anxious and anxious mothers: Infant hearing and language development—ELBW at T2 ↑ than at T3
Kleine et al. (2020) England	Multicenter Longitudinal Cohort Study/Regression analyses	*n* = 140 Mean age 34 y Indicator of mental health: anxiety Mothers were evaluated at T1 = term‐equivalent age T2 = 14 days T3 = 12 m T4 = 22 m (corrected age for preterm infant)	*n* = 140 children (<33 w) Developmental outcome: behavioral and cognitive At 4 to 6 y	↑ mother's trait anxiety at T1/↓ maternal education/↑ gestational age/children's age assessment/↓ children's cognitive development → ↑ children's behavior problems
Wang et al. (2018) China	Longitudinal‐cohort Study/Univariate Analysis of Variance (ANOVA)	*n* = 130 mothers of preterm infants *n* = 130 mothers of full‐term infants Mean age 30 y Indicator of mental health: depression 3 days of delivery	*n* = 130 preterm infants (30 to 36 w) *n* = 130 full‐term infants (37 to 41w) divided into four subgroups: non‐depression‐full‐term, non‐depression‐preterm, depression‐full‐term, and depression‐preterm Developmental outcome: neurodevelopment At 8 m of corrected age	Preterm infants of depressed mothers—↓ children's cognitive and motor development in comparison with the other three groups
Montirosso et al. (2018) Italy	Longitudinal‐multicenter study/ Regression analyses	*n* = 110 Mean age 33 y Indicator of mental health: depression T1 = discharge T2 = 6 m after birth	*n* = 110 Mean gestational age = 29 w Developmental outcome: internalizing and externalizing behavior problems 18 m of corrected age	↑ maternal depressive symptomatology at discharge/↑ infant dysregulation → ↑ infants' internalizing behavior problems
Moea et al. (2016) Norway	Longitudinal‐ prospective/ Between‐group comparison and regression analyses	*n* = 238 mothers of full‐term infants *n* = 64 mothers of preterm infants Mean age 30 y Indicator of mental health: depression 3 m postpartum	*n* = 238 full‐term infants (37 to 42 w) *n* = 64 preterm infants (30 to 36 w) Developmental outcome: social‐emotional behavior T1 = 3 m T2 = 12 m	↑ Maternal depressive symptoms → ↑ later infant problems with social‐emotional functioning at T2
Ren et al. (2024) China	Longitudinal study/Correlation analyses	*n* = 237 mothers of preterm infants Mean age 31 y Indicator of mental health: depression T1 = 40 weeks T2 = 1 m T3 = 3 m T4 = 6 m T5 = 9 m T6 = 12 m (corrected age for prematurity)	*n* = 237 preterm infants Mean age 34 weeks Developmental outcome: physical growth T1 = 40 weeks T2 = 1 m T3 = 3 m T4 = 6 m T5 = 9 m T6 = 12 m	The trajectory of maternal postpartum depression → Slope growth rate of head circumference
Gao et al. (2023) China	Longitudinal study/Correlation analyses	*n* = 318 mothers of preterm infants Mean age 31 y Indicator of mental health: depression T1 = 1 m T2 = 2 m T3 = 3 m T4 = 4 m (corrected age for prematurity)	*n* = 237 preterm infants Mean age 33 weeks Developmental outcome: physical growth T1 = 1 m T2 = 2 m T3 = 3 m T4 = 4 m (corrected age for prematurity)	T1 ‐ ↑ Maternal postpartum depression—↓ growth rate of length T2 and T3 ‐ ↑ Maternal postpartum depression—↓ weight and head circumference
Coletti et al. (2015) Italy	Longitudinal study/Correlation analyses	*n* = 39 mothers of late preterm infants *n* = 40 mothers of very preterm infants Mean age 35 y Indicator of mental health: stress 12 m of premature corrected age	*n* = 39 late preterm infants (33 to 36 w) *n* = 40 very preterm infants (≤32 w) Developmental outcome: neurodevelopment 12 m of corrected age	Very preterm group: ↑ maternal stress concerning child behavior → ↓ child's performance in the language domain, and specifically, receptive language

Abbreviations: ELBW, extremely low birthweight; m, months; VLBW, very low birthweight; w, weeks; y, years.

Regarding the maternal anxiety indicator, when the interaction between maternal anxiety assessed at 3 months of corrected age and birth weight was examined, it was found that motor skills in very low birth weight infants between 9 and 12 months were significantly impacted. Furthermore, hearing and language development in extremely low‐birth‐weight infants, regardless of whether their mothers were anxious or non‐anxious, exhibited poorer performance (Neri et al., 2020). Additionally, maternal trait anxiety assessed at term‐equivalent age, shorter schooling duration, higher GA, and child age assessment can negatively affect the cognitive and behavioral development of preterm children aged four to six (Kleine et al., 2020).

Regarding the maternal depression indicator, preterm children of mothers who were assessed for depressive symptoms 3 days after the preterm birth performed worse at 8 months of corrected age in cognitive and motor development than preterm children of non‐depressed mothers or those born full‐term (Wang et al., 2018). Maternal depressive symptomatology, evaluated at discharge and 6 months postpartum, was linked to greater emotional dysregulation and a higher incidence of internalizing and externalizing behaviors in children at 18 months of corrected age (Montirosso et al., 2018). Additionally, preterm infants, whose mothers displayed depressive symptoms assessed 3 months after delivery, showed declines in social‐emotional functioning at 1 year of age (Moea et al., 2016). Furthermore, the trajectories of postpartum depression appear to be more closely associated with a decline in the growth rate of head circumference, especially in preterm female infants, as well as weight for both genders (Ren et al., 2024; Gao et al., 2023). Maternal stress indicators assessed at 12 months of corrected age, related to the child's behavior, were associated with poorer language performance at 1 year, particularly in receptive language, among very prematurely born infants (Coletti et al., 2015).

Table 2 presents significant findings from studies that evaluated two indicators of maternal mental health and developmental outcomes in preterm children.

**TABLE 2:** Main findings of significant results of studies that assessed two indicators of maternal mental health and developmental outcomes in preterm children (*n* = 2).

Author (year) country	Study design/data analysis	Sample		Main findings
		Mothers	Children	
Santos et al. (2016) EUA	Longitudinal Study/Bivariate analysis and general linear model	*n* = 229 m Mean age 27 y Indicator of mental health: anxiety and stress T1 = at enrollment in the hospital T2 = 2 m T3 = 4 m	*n* = 229 preterm infants (≤ 1750 grams) Developmental outcome: Neurodevelopment 12 m of age corrected for prematurity	↑extreme distress of mothers → ↓ infants’ cognitive and motor development
Pisoni et al. (2020) Italy	Longitudinal Study/Correlation Analysis	*n* = 29 m Mean age 32 y Indicator of mental health: anxiety and stress T1 = during hospitalization T2 = 12 m of corrected age	*n* = 29 preterm infants (<34 w) Developmental outcome: Neurodevelopment T1 = during hospitalization T2 = 12 m of corrected age	↑ several maternal stress symptoms in T1→ ↓ infants’ developmental outcome at T2

Abbreviations: w, weeks; y, years.

Two studies conducted by Santos et al. (2016) and Pisoni et al. (2020) examined how maternal anxiety and stress impact the neurodevelopment of preterm infants at 12 months of corrected age. Both studies found that the more severe the symptoms of maternal distress (anxiety and stress) (Santos et al., 2016) and the more stress symptoms reported by mothers (Pisoni et al., 2020) during hospitalization, the poorer the neurodevelopmental outcomes were (Pisoni et al., 2020), particularly concerning cognitive and motor development (Santos et al., 2016).

Table 3 presents significant findings from studies that evaluated three indicators of maternal mental health and developmental outcomes in preterm children.

**TABLE 3:** Main findings of the significant results of studies that assessed three indicators of maternal mental health and developmental outcomes in preterm children (*n* = 3).

Author (year) country	Study design/data analysis	Sample		Main findings
		Mothers	Children	
Assal‐Zrike et al. (2021) Israel	Longitudinal Study/Bivariate correlation analyses and moderated mediation model	*n* = 48 mothers of preterm infants *n* = 57 mothers of full‐term infants Mean age 27 y Indicators of mental health: anxiety, depression, and distress T1 = 1–4 w after birth T2 = 1 y after birth	*n* = 48 mothers of preterm infants (28‐34 w) *n* = 57 mothers of full‐term infants (>37 w) Developmental outcome: Social responsiveness at 12 m of age	Preterm group: ↑ emotional distress of mothers infants at T1 → ↓ levels of infant social responsiveness ↑ levels of social support at T2 → ↓ levels of maternal emotional distress → ↑ infant social responsiveness
Assal‐Zrike et al. (2022) Israel	Longitudinal Study/Regression analyses	*n* = 48 mothers of preterm infants *n* = 57 mothers of full‐term infants Mean age 27 y Indicators of mental health: anxiety, depression, and distress T1 = shortly after birth T2 = 6 m after birth T3 = 12 m after birth	*n* = 48 mothers of preterm infants (28‐34 w) *n* = 57 mothers of full‐term infants (>37 w) Developmental outcome: Social behavior T2 = 6 m after birth T3 = 12 m after birth	Preterm group: ↑ maternal emotional distress at T2 → ↑ levels of infant social withdrawal at T3
Kenyhercz & Nagy (2020) Hungary	Longitudinal Study/Correlation Analysis	*n* = 27 m of LBW *n* = 40 m of VLBW *n* = 45 m of ELBW Mean age 30 y Indicators of mental health: anxiety, depression, and stress Between 24 and 28 m	*n* = 27 LBW (1500–2499 grams) *n* = 40 VLBW (1000–1499 grams) *n* = 45 ELBW (<1000 grams) Developmental outcome: emotional and behavioral difficulties Between 24 and 28 m	Mothers with ↑ stress,↑ anxiety, ↑ depression symptoms, ↓ satisfaction with life → ↑ child's internalizing‐externalizing behaviors, ↓ quality of life

Abbreviations: ELBW, extremely low birthweight; LBW, low birthweight; m, months; VLBW, very low birthweight; y, years.

Assal‐Zrike et al. (2021) concluded that the more intense the indicators of maternal postpartum emotional distress (a composite score of anxiety and depression indicators) assessed 1 to 4 weeks after birth, the worse the levels of social responsiveness of preterm children at 1 year of age. Furthermore, the authors found that mothers with higher levels of social support when their children were 1 year old had lower levels of emotional distress, resulting in higher infant social responsiveness. Additionally, preterm children had worse outcomes related to social behavior at 12 months after birth in the presence of maternal emotional distress assessed 6 months after birth (Assal‐Zrike et al., 2022). Finally, in the study by Kenyhercz & Nagy (2020), it was possible to identify that the higher the symptoms of maternal anxiety, depression, and stress, evaluated at 24–28 months old of the children, the higher the internalizing and externalizing behaviors in preterm children between the same intervals.

Three studies included in this systematic literature review did not find significant associations between indicators of maternal mental health and developmental outcomes in preterm children. Zengin Akkus and Bahtiyar‐Saygan (2022) compared sleep patterns in preterm and full‐term infants aged 6–18 months, hypothesizing that maternal depression and stress, assessed at 6–18 months of corrected age for prematurity, could affect sleep. Bozkurt et al. (2017) analyzed neurodevelopmental indicators in preterm children between 18 and 22 months of corrected age, correlating them with symptoms of maternal anxiety and depression assessed during the same age range. Finally, Pérez‐Pereira & Baños (2019) compared the behavior of preterm children with that of full‐term children at the age of five, associating it with maternal depression and stress indicators assessed at that same time.

### 3.5 The methodological quality of studies

Regarding the methodological quality of the studies based on the STROBE statement, all studies received an index score of at least 65%, indicating that over half of the applicable items were adequately presented. Additionally, seven studies achieved an index score above 80%, demonstrating excellent methodological quality (Bozkurt et al., 2017; Kleine et al., 2020; Moea et al., 2016; Montirosso et al., 2018; Pisoni et al., 2020; Wang et al., 2018; Zengin Akkus & Bahtiyar‐Saygane, 2022). Six studies recorded an index score between 70% and 80%, indicating good methodological quality (Assal‐Zrike et al., 2022; Coletti et al., 2015; Gao et al., 2023; Neri et al., 2020; Ren et al., 2024; Santos et al., 2016). Three studies had an index score below 70% (Assal‐Zrike et al., 2021; Kenyhercz & Nagy, 2020; Pérez‐Pereira & Baños, 2019).

## 4 DISCUSSION

This systematic review summarizes studies published from 2013 to 2024 that examined the associations between maternal anxiety, depression, stress indicators, and the developmental outcomes of preterm children.

The systematic review revealed a significant association between maternal mental health and the development of preterm children in 75% of the studies analyzed. These findings confirm previous studies highlighting the impact of maternal mental health on their children's developmental outcomes (Field, 2017; Gray et al., 2018; Ionio et al., 2019; Schiavo & Perosa, 2020). However, it is worth noting that most studies have been conducted in high‐income countries.

Regarding the sample of preterm children, a need for uniformity in defining and classifying prematurity was observed. The WHO (World Health Organization, 2023) defines preterm birth as occurring before 37 weeks of GA and offers classifications for preterm neonates as extremely preterm (<28 weeks), very preterm (28 to <32 weeks), and moderate or late preterm (32 to <37 weeks). Furthermore, the analyzed studies revealed variability in the timing of developmental assessments for these children. The studies evaluated developmental outcomes at different ages, with the majority assessing outcomes during the first year of postnatal age. Only one study evaluated long‐term outcomes at 5 to 6 years old. Previous research has shown that preterm birth can have lasting effects on development, persisting in adolescence (Schneider et al., 2014) and adulthood (Husby et al., 2013).

Studies indicate that the hospitalization of preterm neonates in a NICU is an anxiety‐provoking and stressful experience for mothers, even when their health is stable (Field, 2017; Fowler et al., 2019). Furthermore, several studies associate the presence of depressive symptoms with the postpartum experience at different times, ranging from preterm birth (Gerstein et al., 2019; Liu et al., 2017) to short‐ and medium‐term after discharge (Kantrowitz‐Gordon et al., 2016; Liu et al., 2017; Pace et al., 2016). Gerstein et al. (2019) found that mothers of preterm neonates may experience clinically significant depressive symptoms for up to 5 years after birth. This systematic review aims to explore the impact of maternal mental health indicators, such as anxiety, stress, and depression, on the developmental outcomes of preterm infants, where these indicators are highly prevalent. In this way, this review study advances previous reviews that only evaluated the effects of postpartum depression on the developmental outcomes of full‐term children (Aoyagi & Tsuchiya, 2019), the impact of the interaction between mother and child on development, without specifying a sample of preterm children (Rocha et al., 2019), and family outcomes for preterm children (Treyvaud, 2014).

Variability in maternal mental health indicators was observed due to the use of different instruments across varying periods. Regarding the psychometric quality of the instruments used in the studies, they were found to be valid and reliable, appropriate for assessing both maternal mental health. In the 16 studies analyzed, maternal mental health assessments occurred at various times, ranging from immediately after birth or in the first few days, to during hospitalization in the NICU, at discharge, after 1 to 6 months, and in some studies, up to 12 months or 24–28 months after delivery. Symptoms of maternal depression, anxiety, or stress may be associated with difficulties related to different periods experienced by women in the postpartum period. They may peak during neonatal hospitalization and the early postpartum period, possibly decreasing in the first few months, but in some cases reappear or persist longer. A longitudinal cohort study indicated that women who had preterm neonates were at high risk of hospitalization for mental illness in the first 1–2 years after giving birth, but that there were risks that persisted even decades later (Côté‐Corriveau et al., 2022). This variation in the maternal assessment period has significant implications, since maternal distress tends to be more intense in the immediate postpartum period and during periods of maternal or premature hospitalization, tends to fluctuate in the first few months, and can persist long after hospital discharge (Holditch‐Davis et al., 2015). Therefore, the timing of maternal mental health indicator assessment is a critical factor when planning studies and designing interventions or screening programs for maternal mental health in the context of prematurity.

Based on the studies analyzed in this systematic review, the results indicated that most studies found a significant association between indicators of anxiety, depression, and maternal stress and poorer developmental outcomes for preterm children. This association was observed whether it was considered a single indicator of maternal mental health or a combination of two or more indicators. In instances where mothers displayed a single indicator of poor mental health, their children exhibited broad developmental issues, including problems with neurodevelopment, behavior, and social functioning. However, when mothers exhibited multiple indicators, their children were most affected in terms of behavioral outcomes.

The present review showed that anxiety was significantly associated with long‐term behavioral and cognitive impairments in preterm children (Kleine et al., 2020). In this context, Field (2017) demonstrated that maternal anxiety can negatively affect breastfeeding patterns, sleep, mother‐child interaction, and developmental and behavioral indicators of preterm children from infancy to adolescence.

Maternal depressive symptoms were associated with more significant impacts on the neurodevelopment (Wang et al., 2018) and behavior (Moea et al., 2016) of preterm children. Authors have previously characterized maternal depression as a risk factor for maternal responsiveness behaviors and the quality of mother‐infant attachment (Agostini et al., 2014; Gerstein et al., 2019; Santos et al., 2016).

Maternal stress, when linked to indicators of anxiety (Pisoni et al., 2020; Santos et al., 2016) and depression (Assal‐Zrike et al., 2021; 2022), has been shown to worsen the adverse effects on the development of preterm children between the first and second year of postnatal life. These findings support the results found by Gray et al. (2018), which indicated that mothers of infants experienced higher levels of stress than mothers of full‐term infants, potentially contributing to behavioral problems in the first and second year of life.

Therefore, it is crucial to emphasize the significance of psychological evaluation and intervention methods for mothers during the postpartum period, because alleviating symptoms of anxiety, stress, and depression can reduce the negative effects of these factors on both the mother and child. Studies have shown that interventions for mothers of preterm neonates in the NICU mitigate maternal stress and improve mother‐child relationships.

Three studies analyzed in this systematic review did not find significant associations between the indicators of anxiety, depression, and maternal stress and the developmental outcomes of preterm children (Bozkurt et al., 2017; Pérez‐Pereira & Baños, 2019; Zengin Akkus & Bahtiyar‐Saygane, 2022). These studies were cross‐sectional in design, meaning they evaluated indicators of maternal mental health and child development outcomes during the same period, rather than examining the impact of maternal mental health throughout the child's development.

In their study, Bozkurt et al. (2017) found no significant associations between maternal mental health and children's motor development. The authors clarify that although environmental factors, such as stimulation, can play an essential role in motor skill development, the capacity for motor development primarily depends on the maturation of neurological functions. Therefore, maternal depression may not significantly impact the psychomotor development of preterm children. However, regarding the growth rate of infants' length during their first year of life, a slower rate of development is associated with maternal postpartum depression (Gao et al., 2023; Ren et al., 2024). The study by Pérez‐Pereira & Baños (2019) found that children born moderately or late prematurely without biomedical complications do not exhibit more behavioral issues than full‐term children when their mothers have low‐stress symptoms. Finally, Zengin Akkus and Bahtiyar‐Saygane (2022) showed that mothers displayed slight indicators of depression symptoms in their sample, which was not a predictor for changes in the sleep patterns of preterm infants.

All studies scored at least 65% on the STROBE statement for methodological quality, with seven scoring above 80%. This indicates that more than a third of the studies exhibited excellent methodological quality. However, to further enhance their quality, some relevant aspects to consider include calculating sample sizes, addressing missing data, and examining reasons for withdrawal.

## 5 CONCLUSION

In conclusion, this systematic review study demonstrated the association between maternal anxiety, depression, and stress and poorer developmental outcomes in early childhood for preterm children. The presence of multiple indicators of poor maternal mental health increased the risk of negative outcomes in children's neurodevelopmental and behavioral domains.

This review highlights the practical implications that emphasize the importance of maternal mental health on the development of preterm children. It is crucial to assess and address maternal anxiety, depression, and stress, as these factors can affect the growth of preterm neonates. This assessment and intervention should begin during prenatal care and continue through the postpartum period and beyond to promote the mental well‐being of mothers and the healthy development of their children.

It is important to acknowledge that the review has certain limitations. First, most of the studies were conducted in developed and high‐income countries. Therefore, the findings should be treated with caution, since they may not apply to all countries. Besides, none of them reports data on participants' ethnicity or race. The absence of these variables limits the possibility of assessing racial/ethnic disparities or exploring how race/ethnicity may interact with preterm birth, maternal mental health, and developmental outcomes. However, recent publications have demonstrated that race/ethnicity is a critical determinant of perinatal outcomes (Kayode et al., 2024; Rebouças et al., 2024). Second, the definition of prematurity level was not standardized across all studies, which may have impacted the results. Additionally, there was no uniform standard for assessing maternal mental health or the timing of evaluations. Third, there was insufficient control over the confounding variables related to maternal mental health indicators. Fourth, evaluations of developmental outcomes in preterm children primarily focused on the first year of life. Moreover, including studies published within the last 10 years may have limited the findings. Furthermore, the review only includes 16 studies published in the past 10 years, which indicates a lack of scientific research in the area of maternal mental health and the development of preterm children. Finally, the language inclusion criteria may have excluded relevant studies.

## 6 FURTHER RESEARCH

In future studies, it is essential to broaden the sample to include developing countries to improve the generalizability of the results. Furthermore, there should be a standardized definition of prematurity level to more accurately identify potential adverse effects on development. It is also vital to control maternal mental health history to reduce potential sources of bias. Lastly, additional longitudinal studies should be conducted to gather data on the impact of maternal mental health indicators on the long‐term developmental outcomes of preterm children.

## CONFLICT OF INTEREST STATEMENT

The authors declare no conflicts of interest.

## Data Availability

Data sharing is not applicable to this article as no datasets were generated or analyzed during the current study.
